# Epigenetic regulation of cuproptosis in cancer: mechanisms, microenvironment, and therapeutic implications

**DOI:** 10.3389/fcell.2026.1814142

**Published:** 2026-06-09

**Authors:** Xin Zhang, Yixiao Yuan, Lin Tang, Juan Wang, Lincan Duan, Xiulin Jiang, Jia Zhang

**Affiliations:** 1 Comprehensive Ward, Aerospace Center Hospital, Beijing, China; 2 The Third Affiliated Hospital of Kunming Medical University, Peking University Cancer Hospital Yunnan, Kunming, Yunan, China; 3 Department of Thoracic Surgery, Pu’er People’s Hospital, Pu’er, Yunan, China; 4 College of Life Science, University of Chinese Academy of Sciences, Beijing, China; 5 Department of Pulmonary and Critical Care Medicine, Aerospace Center Hospital, Beijing, China

**Keywords:** cancer therapeutics, copper metabolism, cuproptosis, FDX1, metal homeostasis, mitochondrial cell death, proteotoxic stress, TCA cycle

## Abstract

Cuproptosis is a regulated form of cell death triggered by copper overload and dependent on mitochondrial metabolism, particularly through FDX1-mediated protein lipoylation and TCA cycle disruption. Recent studies have revealed that epigenetic mechanisms, including DNA methylation, histone modifications, non-coding RNAs, and RNA chemical modifications such as m^6^A and lactylation, critically regulate the expression of copper transporters, lipoylation enzymes, mitochondrial metabolic proteins, and stress response pathways, thereby modulating tumor cell susceptibility or resistance to cuproptosis. For example, DNA methylation can control copper homeostasis genes through a DNMT/miRNA/copper transporter axis, while histone lactylation links metabolic rewiring to copper accumulation. Non-coding RNAs and RNA modifications fine-tune the transcription and translation of key cuproptosis regulators, providing dynamic control over cell fate. The tumor microenvironment (TME) further influences cuproptosis by shaping copper availability, redox status, hypoxia, and metabolic reprogramming, and interacts with immune surveillance and PD-1/PD-L1 signaling. Translationally, copper ionophores, nanomedicine-based delivery systems, and combination strategies targeting both metabolic vulnerabilities and the TME offer promising approaches to induce tumor-specific cuproptosis while minimizing toxicity. Overall, the integration of molecular, epigenetic, and microenvironmental regulation in cuproptosis provides new insights into tumor metabolic vulnerabilities and offers potential targets for precision anticancer therapies.

## 1 Introduction

Epigenetic modifications comprise a diverse set of reversible chemical processes that regulate gene expression and chromatin architecture without altering the underlying DNA sequence (Sun et al., 2022). These mechanisms, including DNA methylation, histone modifications, non-coding RNAs, and RNA chemical modifications, function in an integrated and highly dynamic regulatory network that governs cell fate decisions, metabolic adaptation, and disease progression (Sun et al., 2022; Ryznar et al., 2021). Their plasticity allows cells to rapidly respond to environmental and metabolic cues, making epigenetic regulation a central determinant of tumorigenesis, cancer progression, and therapeutic response.

Copper (Cu) plays diverse and essential roles in mammalian physiology—including metabolism, cell signaling, differentiation, and regulated cell death (cuproptosis), with recent discoveries revealing novel functions, disease links, and therapeutic potential (Lutsenko et al., 2025). In parallel with advances in epigenetics, the discovery of novel forms of regulated cell death has expanded the conceptual framework of cell fate control. Among these, cuproptosis has recently emerged as a distinct copper-dependent, mitochondria-driven cell death pathway. Mechanistically, excess intracellular copper directly binds to lipoylated components of the tricarboxylic acid (TCA) cycle, triggering aggregation of lipoylated proteins and destabilization of iron–sulfur (Fe–S) cluster proteins, ultimately leading to proteotoxic stress and cell death (Tsvetkov et al., 2022). This process is fundamentally dependent on mitochondrial respiration and metabolic state, distinguishing cuproptosis from other forms of regulated cell death. Recent studies have further identified ferredoxin 1 (FDX1) as a central upstream regulator linking copper metabolism to mitochondrial protein lipoylation. FDX1 promotes protein lipoylation through direct interaction with lipoyl synthase (LIAS), facilitating its functional engagement with the lipoyl carrier protein GCSH and thereby sustaining the activity of key TCA cycle enzymes (Dreishpoon et al., 2023). In addition, FDX1 reduces Cu(II) to Cu(I), enabling copper release from ionophores and directly promoting the lipoylation-dependent cytotoxic process characteristic of cuproptosis (Hsiao et al., 2025). Structural analyses further demonstrate that specific residues within FDX1 are required for both lipoylation and cuproptosis, highlighting a tightly coupled regulatory axis between mitochondrial metabolism, protein lipoylation, and copper-induced cell death (Hsiao et al., 2025). Together, these findings establish a mechanistic core of cuproptosis centered on the FDX1–LIAS–lipoylation–TCA axis. Given that cuproptosis is critically dependent on mitochondrial metabolism, protein lipoylation, and copper homeostasis, it is inherently positioned at the intersection of metabolic regulation and gene expression control. Epigenetic mechanisms may influence cuproptosis through several defined mechanistic entry points, including: i. regulation of copper transport and buffering systems; ii. transcriptional and post-transcriptional control of FDX1 and lipoylation machinery (e.g., LIAS, LIPT1) (Dreishpoon et al., 2023; Hsiao et al., 2025); iii. modulation of TCA cycle enzyme expression and mitochondrial function; and iv. control of cellular stress response pathways that determine tolerance to proteotoxic stress (Tsvetkov et al., 2022).

However, despite rapid progress in defining the biochemical basis of cuproptosis, important gaps remain. Specifically, which epigenetic regulators directly control the FDX1–lipoylation axis remains largely unexplored; how different layers of epigenetic regulation (DNA methylation, histone modification, and RNA regulation) are coordinated in shaping cuproptosis sensitivity is unresolved; and whether epigenetic states causally determine cellular susceptibility to copper-induced proteotoxic stress, or instead act as secondary modulators, remains unclear. In addition, the context-dependent role of tumor microenvironmental factors in epigenetically reprogramming cuproptosis pathways has not been systematically defined. In this mini review, we aim to establish a mechanistically grounded framework linking epigenetic regulation to cuproptosis. We systematically summarize how DNA methylation, histone modifications, non-coding RNAs, and RNA modifications regulate key nodes of the cuproptosis pathway, with a particular focus on the FDX1–lipoylation–mitochondrial metabolism axis. Furthermore, we discuss how tumor microenvironmental factors, mitochondrial metabolic reprogramming, interact with epigenetic networks to modulate cuproptosis sensitivity. By integrating these multilayered regulatory mechanisms, this review seeks to clarify current knowledge gaps and highlight potential therapeutic opportunities targeting the epigenetic control of cuproptosis in cancer.

## 2 Epigenetic regulation of cuproptosis: mechanisms and functional integration

**FIGURE 1:**
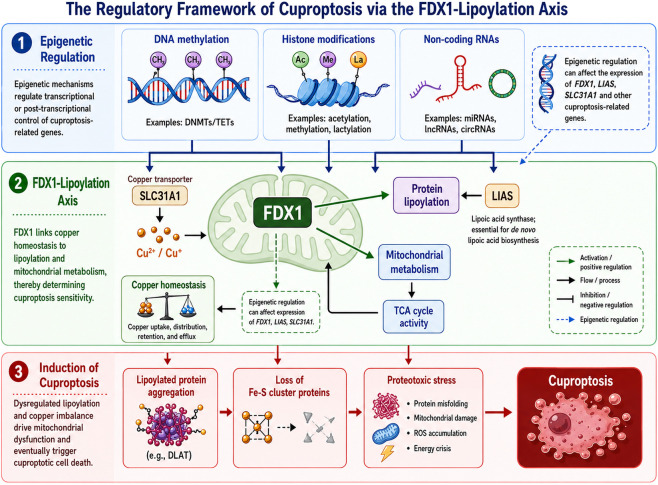
The regulatory framework of cuproptosis via the FDX1-lipoylation axis. This figure illustrates a hierarchical model showing how epigenetic regulation converges on the FDX1-lipoylation axis to determine cellular sensitivity to cuproptosis. In the upper layer, multiple epigenetic mechanisms, including DNA methylation, histone modifications, non-coding RNAs, and RNA modifications, regulate the transcriptional or post-transcriptional expression of cuproptosis-related genes. Representative examples include the DNMT1/miR-302a-3p axis affecting ATP7B expression, H3K18 lactylation promoting Grhl2-dependent SLC31A1 transcription, non-coding RNAs modulating cuproptosis-associated genes, and RNA modifications such as m^6^A, m^5^C, m^7^G, and o8G regulating FDX1, ATOX1, LIPT1, and related lipoylation machinery in a context-dependent manner. In the middle layer, FDX1 acts as a central hub linking copper homeostasis to protein lipoylation and mitochondrial metabolism, thereby influencing TCA cycle activity and cuproptosis susceptibility. SLC31A1 mediates copper uptake, whereas LIAS supports *de novo* lipoic acid biosynthesis required for protein lipoylation. In the lower layer, dysregulated copper homeostasis and aberrant lipoylation lead to lipoylated protein aggregation (e.g., DLAT), loss of Fe-S cluster proteins, proteotoxic stress, mitochondrial dysfunction, reactive oxygen species accumulation, and energy crisis, ultimately resulting in cuproptotic cell death.

Cuproptosis, a copper-dependent form of regulated cell death, is intricately controlled by multiple layers of epigenetic and RNA-mediated regulation (Chen et al., 2022a; Xie et al., 2023). DNA methylation, histone modifications, non-coding RNAs, and RNA chemical modifications converge to modulate the expression, stability, and activity of key copper transporters and cuproptosis effectors (Wang et al., 2025a; Ma et al., 2025; Sun et al., 2023). To provide an integrated overview of these regulatory mechanisms, Figure 1 summarizes how DNA methylation, histone modifications, non-coding RNAs, and RNA modifications converge on the FDX1-lipoylation axis to regulate copper homeostasis, mitochondrial metabolism, and cellular sensitivity to cuproptosis.

### 2.1 DNA methylation: transcriptional control of copper homeostasis and metabolic entry points

DNA methylation is a core epigenetic mechanism that regulates gene transcription through CpG modification mediated by DNA methyltransferases (DNMTs) (Kulis and Esteller, 2010; Jiang et al., 2022). In the context of cuproptosis, its functional importance lies not in general transcriptional repression, but in defining the transcriptional landscape of key upstream determinants, including copper transport systems, mitochondrial metabolism, and lipoylation-related pathways. Emerging evidence indicates that DNA methylation directly regulates copper influx/efflux balance, thereby setting the intracellular copper threshold required to trigger cuproptosis (Long et al., 2021). For example, in hepatocellular carcinoma, plumbagin suppresses DNMT1, leading to hypomethylation and activation of miR-302a-3p, which in turn inhibits the copper exporter ATP7B. This “DNMT1/miR-302a-3p/ATP7B″ axis results in intracellular copper accumulation and induction of cuproptosis (Wang et al., 2025a). This finding establishes DNA methylation as a proximal regulator of copper availability, a key initiating factor of cuproptosis. Beyond copper transport, DNA methylation also regulates genes involved in mitochondrial metabolism and stress responses (Moore et al., 2013), which are essential for cuproptosis execution. Integrative analyses based on TCGA and GEO datasets demonstrate that methylation patterns of cuproptosis-related genes (e.g., CDKN2A, MTF1) correlate with gene expression, tumor stage, and patient survival, and are linked to pathways such as p53 signaling, cell cycle control, and metabolic reprogramming (Long et al., 2021). Notably, these methylation signatures are also associated with immune checkpoint expression and immune infiltration, suggesting that DNA methylation not only modulates cuproptosis sensitivity but also shapes the tumor immune microenvironment (Qin et al., 2023). Collectively, DNA methylation acts at multiple levels, copper transport, metabolic gene expression, and immune regulation, to determine the cellular threshold and systemic context in which cuproptosis can be initiated.

### 2.2 Histone modifications: chromatin-based coupling of metabolic state and copper uptake

Histone modifications dynamically regulate chromatin accessibility and transcriptional programs, thereby linking metabolic state to gene expression (Sun et al., 2018; Zhao and Zhang, 2019; Liu et al., 2022; Liu et al., 2026). In cuproptosis, their key role is to function as metabolic sensors that translate metabolic rewiring into transcriptional control of copper handling and mitochondrial function. Histone acetylation and methylation broadly regulate genes involved in mitochondrial biogenesis, redox homeostasis, and metabolic pathways (Gong and Miller, 2019; Zaib et al., 2022) thereby influencing the metabolic dependency of cells on mitochondrial respiration, a prerequisite for cuproptosis. More importantly, recent studies highlight histone lactylation as a direct mechanistic bridge between metabolic reprogramming and cuproptosis. Under high-lactate conditions, H3K18 lactylation is increased, promoting transcription of the factor Grhl2, which subsequently activates the copper transporter SLC31A1 (CTR1), leading to enhanced copper uptake and induction of cuproptosis (Shen et al., 2024). Silencing SLC31A1 significantly attenuates lactate-induced cuproptosis, demonstrating that this pathway directly links metabolic byproducts to copper-dependent cell death. This mechanism illustrates a clear conceptual advance: metabolite-driven histone modifications can directly regulate copper transport capacity, thereby coupling metabolic states (e.g., glycolytic/lactate-rich environments) to cuproptosis susceptibility. Thus, histone modifications may provide a regulatory link between metabolic rewiring, chromatin-state changes, and copper-induced cytotoxicity, rather than functioning as a universally central integrative layer in cuproptosis.

### 2.3 Non-coding RNAs: network-level regulation of FDX1 axis and cuproptosis sensitivity

#### 2.3.1 FDX1 regulation

Non-coding RNAs (ncRNAs) regulate gene expression through post-transcriptional, transcriptional, and epigenetic mechanisms (Gao et al., 2023; Hombach and Kretz, 2016; Chen et al., 2022b; Xiong et al., 2021). In cuproptosis, their primary function is to orchestrate network-level control of key regulatory nodes, particularly the FDX1–lipoylation axis, copper transport, and metabolic adaptation. A prominent example is the lncRNA PVT1, which directly binds to the FDX1 promoter, promotes H3K27ac deposition, and recruits the transcription factor SF1, thereby enhancing FDX1 transcription. This activation increases protein lipoylation-dependent proteotoxic stress and induces cuproptosis (Ma et al., 2025). This finding is particularly important because it positions ncRNAs as direct upstream regulators of the core cuproptosis machinery (FDX1). FDX1 can also be regulated through a ceRNA-dependent mechanism. In hepatocellular carcinoma, FDX1 expression is downregulated, whereas higher FDX1 expression is associated with better prognosis and increased sensitivity to oxaliplatin. Mechanistically, LINC02362 acts as a competing endogenous RNA that binds hsa-miR-18a-5p, thereby relieving miR-18a-5p-mediated repression of FDX1. Activation of the LINC02362/hsa-miR-18a-5p/FDX1 axis suppresses HCC cell proliferation and enhances oxaliplatin-induced cuproptosis. In contrast to FDX1-activating ncRNAs, some ncRNAs suppress FDX1 expression and thereby inhibit cuproptosis (Xu et al., 2025a). In high-grade serous ovarian cancer, the lncRNA RP11-199F11.2 is highly expressed and correlates with advanced FIGO stage and lymph node metastasis. Mechanistically, RP11 is proposed to bind the 3′-UTR of FDX1, reducing FDX1 translation and limiting copper death (Xu et al., 2025a). Functional studies showed that modulation of RP11 affects tumor growth, while treatment with the copper ionophore elesclomol-Cu restores FDX1 expression, reactivates copper death, and markedly suppresses tumor growth without obvious histological toxicity (Xu et al., 2025a). CircRNAs may also participate in FDX1 regulation (Xu et al., 2025b). In lung cancer, ROS-induced oxidative modification of circKIAA1797 promotes its recognition by the reader protein YBX1, thereby increasing circKIAA1797 stability and cytoplasmic accumulation. Functionally, circKIAA1797 promotes lung cancer progression both *in vitro* and *in vivo* (Xu et al., 2025b). Mechanistically, circKIAA1797 inhibits cuproptosis by directly binding FDX1 mRNA, reducing its stability and suppressing FDX1 expression. In parallel, circKIAA1797 interacts with STAT1 protein and inhibits LIPT1 transcription, further weakening the lipoylation machinery required for cuproptosis (Xu et al., 2025b). Together, these studies indicate that ncRNAs can either promote or inhibit cuproptosis depending on their effects on FDX1. FDX1-activating ncRNAs, such as PVT1 and LINC02362, enhance copper-dependent proteotoxic stress and may improve sensitivity to copper ionophores or chemotherapy. In contrast, FDX1-suppressive ncRNAs, including RP11-199F11.2 and circKIAA1797, attenuate cuproptosis and promote tumor progression. Therefore, the ncRNA–FDX1 regulatory network represents a key epigenetic and post-transcriptional layer controlling cuproptosis sensitivity and may provide new therapeutic opportunities for cuproptosis-based cancer treatment.

#### 2.3.2 Copper transports

Copper transporters are essential regulators of intracellular copper homeostasis and therefore influence cuproptosis sensitivity (Guo et al., 2023). In addition to classical copper transporters such as SLC31A1/CTR1, ATP7A, and ATP7B (Guo et al., 2023), emerging evidence suggests that ncRNAs and RNA modification regulators can modulate copper transport-related pathways and thereby affect tumor progression and therapeutic response (Howell et al., 2010). In breast cancer, the lncRNA Z68871.1 links cuproptosis with m^6^A modification and immune regulation through the RBM15/YTHDC2/ATP7A axis. Mechanistically, the m^6^A writer RBM15 and reader YTHDC2 regulate Z68871.1-associated signaling, which affects ATP7A-mediated copper transport, tumor progression, and the immune microenvironment. This finding suggests that copper transporter regulation may be integrated with RNA epitranscriptomic control rather than functioning as an isolated metal transport process (Zhang et al., 2025). Similarly, LINC00607 contributes to cuproptosis resistance by interacting with PDHA1, a key mitochondrial metabolic regulator. Although this mechanism is not limited to copper transport itself, it connects copper-induced cell death with mitochondrial metabolism and PD-L1-mediated immune evasion. Therefore, LINC00607 provides another example of how ncRNA-mediated regulation can coordinate copper homeostasis, metabolic adaptation, and immune escape in cancer (Lu et al., 2025). Together, these findings indicate that copper transporters are not only passive regulators of intracellular copper levels but also components of broader regulatory networks involving ncRNAs, m^6^A modification, mitochondrial metabolism, and tumor immunity. This regulatory complexity may partly explain the context-dependent sensitivity of tumors to cuproptosis-based therapies.

#### 2.3.3 Mitochondrial metabolism

Cuproptosis preferentially occurs in cells that depend on mitochondrial respiration and an active tricarboxylic acid cycle, indicating that mitochondrial metabolic status is a critical determinant of copper-induced cell death. Additional ncRNAs, including circFRMD4A, circSpna2, and miR-185-5p, regulate copper transporters, mitochondrial metabolism, and oxidative stress pathways, thereby influencing cuproptosis and clinical outcomes (Liao et al., 2025). A recent study identified circFRMD4A as an important mediator linking p53-dependent metabolic reprogramming to cuproptosis. circFRMD4A is derived from the FRMD4A transcript, which is transcriptionally activated by p53, while its circularization is facilitated by the RNA-binding protein EWSR1 (Liao et al., 2025). Functionally, circFRMD4A acts as a tumor suppressor and enhances cancer cell sensitivity to elesclomol-induced cuproptosis. Mechanistically, circFRMD4A interacts with and inactivates PKM2, a key glycolytic enzyme, thereby reducing lactate production and redirecting glycolytic flux toward the tricarboxylic acid cycle (Liao et al., 2025). This metabolic shift increases mitochondrial dependence and sensitizes tumor cells to copper-induced toxicity. Importantly, combined treatment with p53 agonists and elesclomol suppresses tumor growth in xenograft models, suggesting that restoration of p53–circFRMD4A signaling may provide a therapeutic strategy for enhancing cuproptosis in cancers with wild-type p53. In addition to tumor metabolism, ncRNA-mediated regulation of mitochondrial oxidative stress and copper transport may also influence cuproptosis-related pathological outcomes. In traumatic brain injury, circSpna2 expression is decreased and negatively associated with depression-like symptoms. Mechanistically, circSpna2 binds the ubiquitin ligase Keap1, thereby modulating the Nrf2/Atp7b signaling axis. Since ATP7B is a key copper transporter involved in copper efflux and intracellular copper homeostasis, circSpna2 may regulate cuproptosis by coordinating oxidative stress defense with copper transport. Functionally, circSpna2 overexpression alleviates cuproptosis after traumatic brain injury through the Keap1/Nrf2/Atp7b pathway, whereas circSpna2 knockdown aggravates depression-like phenotypes (Du et al., 2024a). Although this evidence is derived from a neurological injury model rather than cancer, it supports the broader concept that ncRNAs can regulate cuproptosis by controlling copper transporter expression and mitochondrial redox balance (Du et al., 2024a). These studies highlight mitochondrial metabolism as a key ncRNA-regulated vulnerability that may be exploited to improve cuproptosis-based therapeutic strategies.

### 2.4 RNA modifications: epitranscriptomic control of FDX1–Lipoylation machinery and dynamic response

RNA modifications, particularly m^6^A, provide a rapid and reversible mechanism for regulating mRNA fate, including stability, translation, and localization (Jiang et al., 2021; Cerneckis et al., 2023; Chen et al., 2025; Wang et al., 2025b; Wang et al., 2025c). In cuproptosis, these modifications primarily act at the post-transcriptional level to control key effectors, especially FDX1 and lipoylation-related genes. Multiple studies demonstrate that m^6^A modification directly regulates FDX1 expression and function. In gastric cancer, METTL16-mediated m^6^A modification of FDX1 mRNA is further modulated by SIRT2-dependent lactylation, influencing sensitivity to the copper ionophore elesclomol (Sun et al., 2023). In hepatocellular carcinoma, METTL3 suppresses FDX1 translation via FMR1, conferring resistance to cuproptosis, whereas its inhibition enhances sensitivity to Elesclomol-Cu treatment (Jiang et al., 2026). Similarly, METTL14 and ALKBH5 regulate FDX1 or copper chaperone ATOX1 mRNA stability, affecting cuproptosis in ischemic injury and leukemia (Ding et al., 2025; Peng et al., 2026). In addition to FDX1, RNA modifications also regulate lipoylation machinery components. For example, YTHDF2-mediated m^6^A recognition promotes degradation of LIPT1, and restoration of LIPT1 enhances cuproptosis and induces ER stress (Du et al., 2024b). These findings highlight that m^6^A modification controls both upstream regulators (FDX1) and downstream executors (lipoylation enzymes). Therefore, m^6^A regulation should not be regarded as a universally dominant mechanism in cuproptosis, but rather as a context-dependent regulatory layer whose functional impact may vary according to tumor type, metabolic state, copper availability, and the expression pattern of specific m^6^A writers, erasers, and readers. Beyond m^6^A, other RNA modifications expand the regulatory landscape. m^5^C modification mediated by NSUN5 stabilizes GLS mRNA, promoting metabolic rewiring and cuproptosis resistance in cholangiocarcinoma. In lung cancer, oxidative RNA modification (o8G) stabilizes circKIAA1797, which suppresses FDX1 and LIPT1 expression, thereby inhibiting cuproptosis (Xu et al., 2025b). Additionally, m^7^G methyltransferase Mettl1 protects against FDX1-mediated copper toxicity in septic cardiomyopathy (Siang et al., 2026). Together, RNA modifications establish a dynamic epitranscriptomic layer that rapidly adjusts the expression of key cuproptosis regulators in response to metabolic and stress signals. This layer is uniquely positioned to fine-tune the FDX1, lipoylation–mitochondrial metabolism axis, thereby determining cellular sensitivity to copper-induced proteotoxic stress.

## 3 Cuproptosis in immunogenic signaling and cancer immunotherapy

### 3.1 Cuproptosis in immunogenic signaling and immune cell infiltration

Emerging evidence indicates that cuproptosis is not only a mitochondria-centered form of regulated cell death but also a critical modulator of tumor immunogenicity and immune microenvironment remodeling (Tong et al., 2022). Through its dependence on mitochondrial metabolism, protein lipoylation, and copper homeostasis, cuproptosis is uniquely positioned to influence immunogenic signaling pathways and immune cell infiltration, thereby shaping tumor progression and therapeutic responses. Pan-cancer analyses have revealed that cuproptosis-related genes (CRGs) are widely dysregulated across tumor types and are closely associated with immune-related features (Qin et al., 2023; Li et al., 2024a; Ge et al., 2026; Long et al., 2022). Notably, cuproptosis scores (CS) are significantly correlated with immune checkpoint molecules such as PD-L1, tumor mutational burden (TMB), and microsatellite instability (MSI), suggesting a strong link between cuproptosis and tumor immune escape mechanisms. Furthermore, CS is negatively correlated with TME scores in multiple cancers, indicating that increased cuproptosis activity may be associated with reduced immune cell infiltration or an immunosuppressive microenvironment. Single-cell transcriptomic analyses further demonstrate that CRG activity is enriched in malignant cells, reinforcing the role of cuproptosis in tumor-intrinsic immune regulation (Qin et al., 2023).

Mechanistically, cuproptosis can directly induce immunogenic cell death (ICD), thereby promoting antitumor immune responses. For instance, copper ionophore-based nanoplatforms that induce cuproptosis in triple-negative breast cancer (TNBC) cells not only trigger mitochondrial dysfunction and proteotoxic stress but also lead to ICD characterized by immune activation. These processes are often accompanied by intracellular copper accumulation through downregulation of ATP7B and aggregation of lipoylated proteins such as DLAT, ultimately activating both apoptosis and cuproptosis pathways and enhancing immune system engagement (Wang et al., 2024a). Importantly, the tumor microenvironment plays a decisive role in modulating the immunogenic effects of cuproptosis. Hypoxia, a hallmark of many solid tumors, suppresses the expression of key cuproptosis regulator FDX1, thereby limiting the efficacy of copper-dependent cell death. Targeting hypoxia through nanotechnology-based strategies can restore mitochondrial metabolism and enhance cuproptosis, which in turn promotes ICD and strengthens antitumor immunity. For example, ROS-responsive nanomedicine (CuET@PHF) has been shown to overcome hypoxia, induce cuproptosis in cancer stem cells (CSCs), and simultaneously activate immune responses, leading to reduced tumor recurrence and metastasis (Xiao et al., 2025). This finding highlights a critical link between metabolic reprogramming, cuproptosis activation, and immune surveillance.

At the signaling level, cuproptosis is closely associated with activation of innate immune pathways, particularly the cGAS–STING axis, which senses cytosolic DNA and initiates type I interferon responses. Mitochondrial damage induced by cuproptosis can lead to the release of mitochondrial DNA (mtDNA), thereby activating cGAS–STING signaling and promoting antitumor immunity (Chen et al., 2024). For instance, mitochondria-targeted nanoassemblies (TCe6@Cu/TP5 NPs) induce cuproptosis and reactive oxygen species (ROS) accumulation, resulting in activation of AMPK signaling, degradation of PD-L1, and stimulation of the cGAS–STING pathway. This cascade enhances dendritic cell (DC) maturation and T cell activation, thereby amplifying systemic antitumor immune responses (Chen et al., 2024). Similarly, photothermal-enhanced cuproptosis strategies further demonstrate how mitochondrial dysfunction can reshape immune responses. Copper-based nanoplatforms (CZP NPs) induce oxidative stress, disrupt mitochondrial integrity, and trigger mtDNA release, which synergizes with metal ion signaling to robustly activate the cGAS–STING pathway. This activation reverses the immunosuppressive TME, enhances PD-L1 expression, and sensitizes tumors to immune checkpoint blockade therapy, particularly anti-PD-L1 treatment (Zhou et al., 2025). These findings suggest that cuproptosis not only initiates immune signaling but can also reprogram immune checkpoint dynamics, thereby influencing immunotherapy efficacy. Collectively, current evidence supports a model in which cuproptosis acts as a bridge between mitochondrial metabolic stress and immune activation. Through induction of immunogenic cell death, modulation of immune checkpoints, and activation of innate immune pathways such as cGAS–STING, cuproptosis plays a multifaceted role in shaping tumor immunogenicity and immune cell infiltration (Zhou et al., 2025). Moreover, its sensitivity to metabolic conditions (e.g., hypoxia) and epigenetic regulation further underscores its context-dependent role within the tumor microenvironment. Targeting cuproptosis, particularly in combination with immunotherapy, represents a promising strategy to overcome immune resistance and enhance antitumor efficacy.

### 3.2 Cuproptosis and cGAS-STING activation

Cuproptosis-associated cGAS-STING activation should be interpreted cautiously, because current evidence does not yet establish this pathway as a strictly cuproptosis-specific immune response (Yan et al., 2024; Li et al., 2025; Bai et al., 2025). Several recent nanomedicine studies suggest that copper-based therapeutic systems can induce mitochondrial damage, mtDNA release, and subsequent cGAS-STING activation, thereby enhancing innate and adaptive antitumor immunity (Yan et al., 2024; Li et al., 2025; Bai et al., 2025). For example, zinc-copper bimetallic peroxide nanoparticles were reported to promote Cu^2+^ release, DLAT aggregation, loss of iron-sulfur cluster proteins, and cuproptosis, while Zn^2+^/ROS-induced mitochondrial injury facilitated mtDNA release and cGAS-STING activation (Liu et al., 2025). Similarly, Cu-ZnO_2_@PDA nanoplatforms induced mitochondrial disruption, mtDNA release, and cGAS-STING signaling in triple-negative breast cancer, thereby improving anti-PD-L1 responsiveness (Zhou et al., 2025). Other studies also linked cuproptosis-based nanotherapeutics with STING pathway activation and improved antitumor immunity, particularly through enhanced dendritic cell maturation, macrophage polarization, and T-cell infiltration (Yan et al., 2024; Li et al., 2025; Bai et al., 2025). More direct mechanistic evidence was provided by copper complexes such as Cu-DPPZ-Py^+^ and Cu-Elesclomol, which induced cuproptosis-associated mtDNA release and cGAS-STING activation, whereas apoptosis induced by a structurally related copper complex showed distinct immunological consequences (Zhu et al., 2024). Nevertheless, because many of these systems simultaneously induce ROS production, mitochondrial stress, metal-ion release, ferroptosis-like injury, or nanomaterial-mediated immune stimulation, cGAS-STING activation should currently be viewed as a mitochondrial-damage-associated immune consequence of copper-based cytotoxicity rather than a fully defined cuproptosis-specific pathway. Further studies are needed to determine whether FDX1-dependent protein lipoylation, DLAT aggregation, or iron-sulfur cluster loss directly and selectively triggers cGAS-STING signaling in endogenous cuproptosis models.

### 3.3 Therapeutic strategies linked to epigenetic regulation, mitochondrial dependency, and cuproptosis-specific mechanisms

Therapeutic strategies targeting cuproptosis should be interpreted in relation to their underlying mechanisms, including copper transport, mitochondrial metabolism, protein lipoylation, oxidative stress, and epigenetic or epitranscriptomic regulation. Rather than simply functioning as copper delivery approaches, these interventions may exploit specific vulnerabilities in the cuproptosis machinery. Radiotherapy has been shown to induce cuproptosis by increasing mitochondrial copper accumulation. Mechanistically, radiotherapy upregulates CTR1/SLC31A1 and depletes mitochondrial glutathione, thereby promoting copper-dependent mitochondrial toxicity (Lei et al., 2025). This process is accompanied by depletion of lipoylated proteins and iron–sulfur cluster proteins, which are key features of cuproptosis. In radioresistant tumors, reduced BACH1 expression derepresses metallothioneins such as MT1E/MT1X, limiting copper toxicity. Thus, combining copper ionophores with radiotherapy may overcome resistance by restoring cuproptosis sensitivity (Lei et al., 2025). Copper ionophore-based combinations also connect cuproptosis with immune regulation. In NSCLC cells, disulfiram (DSF) increases ATP7B and PD-L1, suggesting adaptive copper efflux and immune escape (Li et al., 2024b). Combining DSF with PD-L1 or HIF-1α inhibition enhances oxidative stress, increases FDX1 and SLC31A1, and suppresses ATP7B, PD-L1, and HIF-1α, thereby strengthening cuproptosis-associated cytotoxicity (Li et al., 2024b). Mitochondrial metabolism and ncRNA regulation further influence therapeutic responses. In HCC, the PDHA1–LINC00607 complex promotes tumor progression (Lu et al., 2025), cuproptosis resistance, and PD-L1-mediated immune escape (Lu et al., 2025). Targeting this axis may therefore enhance copper-induced cell death while improving antitumor immunity. In pancreatic cancer, SERPINB3 suppresses FDX1 transcription through MAPK signaling and promotes PD-L1 expression, leading to both cuproptosis resistance and immune evasion. Based on this mechanism (Huang et al., 2025), combined MAPK inhibition, cuproptosis induction, and anti-PD-1 therapy may provide a rational therapeutic strategy, especially in SERPINB3-high tumors (Huang et al., 2025). Epitranscriptomic regulation also provides a mechanism-based therapeutic entry point. In colorectal cancer, NAT10-mediated ac4C modification stabilizes DLAT mRNA, while NAT10 lactylation enhances its catalytic activity and promotes DLAT-dependent cuproptosis. The combination of elesclomol and the SIRT1 inhibitor selisistat enhances cuproptosis by reinforcing NAT10 lactylation and DLAT expression (Yang et al., 2026). Together, these studies suggest that cuproptosis-based therapies should be linked to defined molecular mechanisms, including copper transporter regulation, mitochondrial metabolic dependency, FDX1/DLAT-centered cuproptosis machinery, immune checkpoint regulation, and RNA modification-mediated control.

## 4 Discussion and perspectives

Cuproptosis, a regulated cell death dependent on mitochondrial metabolism and copper imbalance, provides a novel framework for understanding tumor metabolic vulnerabilities (Xie et al., 2023). At the molecular level, cellular sensitivity to cuproptosis depends on multiple key determinants, including the expression of copper uptake and efflux systems, TCA cycle activity, abundance of lipoylated proteins, proteostasis capacity, and potentially epigenetic regulation of these pathways. Tumor subtypes reliant on oxidative phosphorylation are typically more sensitive to cuproptosis, whereas highly glycolytic tumors may be relatively resistant. Enzymes regulating lipoylation, iron–sulfur cluster stability, and stress response pathways are critical for cuproptosis execution (Lutsenko et al., 2025).

### 4.1 Epigenetic regulation and knowledge gaps

Emerging evidence suggests that epigenetic mechanisms, including DNA methylation, histone modifications, and non-coding RNAs, may influence the expression of copper transporters, lipoylation enzymes, and stress response genes, thereby modulating cuproptosis susceptibility. However, the precise roles of these epigenetic layers in the context of cuproptosis remain largely undefined. Key knowledge gaps include: Which epigenetic modifications directly regulate copper homeostasis genes and mitochondrial stress responses. How epigenetic landscapes interact with TME cues, such as hypoxia, inflammation, or acidity, to influence cuproptosis thresholds. Whether epigenetic modulators can be therapeutically targeted to sensitize tumors to cuproptosis without harming normal tissues. Addressing these questions requires integrated frameworks that link epigenetic regulation, copper metabolism, mitochondrial adaptation, and proteostasis to cuproptosis outcomes. Such frameworks would allow testable hypotheses for the interplay between intrinsic molecular states and extrinsic TME signals in determining tumor vulnerability.

### 4.2 Tumor microenvironment interactions

TME plays a central role in modulating copper availability and the activation of cuproptosis (Mao et al., 2021). Microenvironmental factors such as hypoxia, acidic pH, and oxidative stress can profoundly influence mitochondrial metabolism, alter copper redox states, and limit the availability of lipoylated TCA cycle proteins required for cuproptosis (Mao et al., 2021). In addition, stromal cells, cancer-associated fibroblasts (CAFs), endothelial cells, and various immune populations can secrete metal-binding proteins, modulate copper transporters, or regulate ion channels, dynamically shaping local copper distribution and bioavailability. Metabolic competition between tumor and immune cells may further affect copper uptake and utilization, creating spatial heterogeneity in cuproptosis susceptibility (Xia et al., 2021). These microenvironmental interactions can also impact the expression and activity of key regulators such as FDX1, LIAS, and METTL16, thereby influencing both the initiation and propagation of copper-induced cell death. Understanding these TME-mediated mechanisms is critical not only for explaining heterogeneous cuproptosis responses across tumor regions and cellular subpopulations but also for guiding therapeutic strategies that combine copper ionophores with agents targeting hypoxia, redox balance, or stromal support, ultimately enhancing the efficacy and specificity of cuproptosis-based interventions.

### 4.3 Distinguishing cuproptosis from other programmed cell deaths

#### 4.3.1 Interconnections between epigenetic regulation in differ cell death

Epigenetic modifications, including DNA methylation, histone modifications, non-coding RNAs, and m^6^A methylation, are involved in regulating multiple forms of programmed cell death, revealing a complex network of interconnections (Jiang et al., 2023; Zhou et al., 2024). Shared molecular regulators, such as SLC7A11, ROS, and p53, are subject to epigenetic control and influence ferroptosis, cuproptosis, disulfidptosis, apoptosis, autophagy, and pyroptosis (Zhou et al., 2024). For example, DNA methylation and histone modifications modulate SLC7A11 expression, thereby affecting both ferroptosis and disulfidptosis (Zhou et al., 2024). ROS levels, calcium signaling, and stress response pathways act as common mediators that link different death pathways, with epigenetic modifications fine-tuning their activity (Zhou et al., 2024). Furthermore, lncRNAs and m^6^A modifications can simultaneously impact multiple death modalities, integrating metabolic cues, stress responses, and TME signals to coordinate cellular fate decisions.

#### 4.3.2 Distinct epigenetic features across cell death modalities

Despite these interconnections, each cell death mode exhibits unique epigenetic regulatory mechanisms. Ferroptosis is tightly regulated by methylation and m^6^A modifications of genes such as SLC7A11, ACSL4, and FTH1, modulating iron and lipid metabolism (Damiescu et al., 2024). Pyroptosis is primarily influenced by epigenetic regulation of inflammasome components (NLRP3, AIM2) and Gasdermin proteins, with DNA methylation and m^6^A modification determining the activation of caspase pathways (Damiescu et al., 2024). Cuproptosis, a recently defined form of copper-dependent death, appears to be regulated by lncRNA-mediated modulation of mitochondrial metabolism and metal-binding proteins, while m^6^A methylation of FDX1 promotes cuproptosis under copper stress (Zhou et al., 2024). Disulfidptosis involves lncRNA networks that influence redox balance and actin cytoskeleton integrity, yet other epigenetic contributions remain largely unexplored (Zhou et al., 2024). These distinct features highlight that, although overlapping regulators exist, each death modality has specific epigenetic signatures that define its susceptibility, execution, and interaction with the tumor microenvironment.

### 4.4 Translational considerations of copper ionophore therapy

Copper ionophores, such as elesclomol and disulfiram, have emerged as promising agents to selectively induce cuproptosis in cancer cells by elevating intracellular copper levels and directly targeting mitochondrial metabolism (Wang et al., 2023). Copper and cuproptosis play key roles in cancer, and drugs that modulate intracellular copper, such as elesclomol (ES), disulfiram (DSF), chloroquine (CQ), and 8-hydroxyquinoline (8-OHQ),offer potential for therapy (Cui et al., 2026). Elesclomol (ES), initially developed as an oxidative stress–inducing antitumor agent, functions as a copper ionophore and has shown an acceptable safety profile in certain clinical settings; however, its tolerability and therapeutic window may be disease- and context-dependent, and systematic clinical evaluation of the ES–Cu complex remains limited (Cui et al., 2026). DSF, long used for alcohol dependence, shows some efficacy in glioblastoma when combined with copper, especially in BRAF-mutant cases, but overall clinical results remain limited (Wang et al., 2024b). Major challenges include maintaining high tumor copper levels and minimizing toxicity. Future research should focus on targeted delivery, combination therapies, and strategies for effective clinical translation of cuproptosis-based cancer treatments (Wang et al., 2024b). Despite their potential, translational application requires careful evaluation of toxicity, therapeutic window, and treatment context. Copper is an essential cofactor for many cellular processes, but excess copper triggers proteotoxic stress through direct binding to lipoylated TCA cycle proteins, loss of iron-sulfur cluster proteins, and activation of stress responses, which can induce cuproptosis (Tsvetkov et al., 2022). Critical regulators such as FDX1, LIAS, and METTL16 modulate the sensitivity of tumor cells to copper overload, and their expression may define patient-specific therapeutic thresholds. Additionally, the tumor microenvironment, ncluding local copper availability, metabolic state, and lactate levels, can significantly influence cuproptosis efficacy, highlighting the importance of context-dependent dosing strategies (Tsvetkov et al., 2017). Preclinical studies suggest that copper ionophores can be effectively combined with metabolic modulators, SIRT2 inhibitors, or conventional therapies to enhance anti-tumor effects while mitigating toxicity to normal tissues. Overall, understanding copper homeostasis, metabolic dependencies, and molecular determinants of sensitivity will be crucial to optimizing the therapeutic window and developing rational combination strategies for clinical translation of copper-based anticancer therapies. Future research on nanomaterial-induced cuproptosis should focus on three areas: first, deepening mechanistic understanding of copper homeostasis, immune cell cuproptosis, tumor microenvironment interactions, and crosstalk with other cell death pathways; second, advancing technologies for imaging copper metabolism and delivering copper-based nanomaterials precisely; and third, developing copper-free nanomaterials that activate endogenous cuproptosis to enable safer and more effective immunotherapy.

### 4.5 Translational challenges and potential clinical limitations of copper ionophore therapy

Despite the strong mechanistic rationale for using copper ionophores to induce cuproptosis, their clinical translation requires cautious evaluation (Liu et al., 2023). The safety of elesclomol and related copper carriers should not be interpreted as universally favorable, because tolerability may vary depending on tumor type, disease stage, mitochondrial metabolic status, systemic copper homeostasis, liver function, and combination treatment regimens (Liu et al., 2023). In addition, patients with altered copper metabolism or impaired detoxification capacity may be more vulnerable to copper-associated toxicity (Liu et al., 2023). Therefore, the statement that ES has a “promising safety profile” should be considered context-dependent rather than broadly generalizable. Several limitations may also explain the potential clinical failure of copper ionophore-based therapy (Rieber, 2020). First, not all tumors are equally sensitive to cuproptosis, as copper-induced cell death appears to depend on mitochondrial respiration, TCA cycle activity, protein lipoylation, and the expression of key regulators such as FDX1, LIAS, DLAT, and LIPT1. Tumors with strong glycolytic dependence, low mitochondrial activity, or reduced lipoylation machinery may be intrinsically resistant (Rieber, 2020). Second, copper ionophores may induce non-specific oxidative stress or mitochondrial damage, making it difficult to distinguish cuproptosis-specific effects from broader cytotoxicity (Rieber, 2020). Third, biomarkers for patient selection remain insufficiently established. Without reliable indicators of copper dependency, mitochondrial metabolic state, or FDX1–lipoylation axis activity, clinical responses may be heterogeneous (He et al., 2025). Finally, the optimal dosing strategy, copper supplementation status, combination partners, and toxicity management remain unclear, especially for ES–Cu complexes, which have not yet been systematically evaluated in clinical studies (He et al., 2025). Together, these considerations suggest that copper ionophore therapy should be developed through a biomarker-guided and context-specific strategy (He et al., 2025). Future studies should define tumor types most likely to respond, identify predictive biomarkers of cuproptosis sensitivity, evaluate the pharmacokinetics and safety of ES–Cu complexes, and clarify whether combination strategies with chemotherapy, immunotherapy, metabolic inhibitors, or epigenetic drugs can improve therapeutic efficacy while minimizing toxicity.

## 5 Conclusion

Cuproptosis provides a new conceptual framework for understanding how copper metabolism, mitochondrial respiration, protein lipoylation, and proteotoxic stress jointly regulate tumor cell fate. This review highlights that cuproptosis is not an isolated metabolic death program, but is tightly controlled by multilayered epigenetic mechanisms, including DNA methylation, histone modifications, non-coding RNAs, and RNA modifications. These regulatory layers converge on key cuproptosis-related nodes, such as copper transporters, FDX1, LIAS, LIPT1, TCA cycle enzymes, and mitochondrial stress-response pathways, thereby shaping cellular sensitivity to copper-induced cytotoxicity. In particular, the FDX1–lipoylation–mitochondrial metabolism axis appears to represent a central hub through which epigenetic and metabolic signals determine cuproptosis susceptibility. Importantly, cuproptosis is closely linked to the tumor microenvironment and antitumor immunity. By inducing mitochondrial dysfunction, immunogenic cell death, cGAS–STING activation, and immune checkpoint modulation, cuproptosis may enhance tumor immunogenicity and improve responses to immune checkpoint blockade. However, hypoxia, metabolic heterogeneity, copper buffering systems, and epigenetic plasticity may limit its therapeutic efficacy. Therefore, future studies should define the causal relationships between epigenetic states and cuproptosis sensitivity, identify reliable biomarkers for patient stratification, and develop safer delivery systems for copper ionophores or cuproptosis-inducing nanomedicines. Rational combinations with epigenetic drugs, metabolic modulators, radiotherapy, ferroptosis inducers, or immunotherapy may further expand the therapeutic potential of cuproptosis. Overall, targeting the epigenetic regulation of cuproptosis offers a promising strategy to exploit tumor metabolic vulnerabilities and overcome therapy resistance in cancer.
